# Supplementary material for: Resolving the full spectrum of human genome variation using Linked-Reads
Source: Genome Res. 2019 Apr;29(4):635–45. doi: 10.1101/gr.234443.118 (PMC6442396; doi:10.1101/gr.234443.118)
Supplement: Supplemental Material [file supp_gr.234443.118_Supplemental_longranger-2.2.zip › longranger-2.2.tar.bz2!/lr2.2-public/lib/go/src/code.google.com/vitess/docs/user-guide/sharding.html]

Vitess / Sharding


Toggle navigation

- Overview
- Guides
- Reference
- Blog
- GitHub

- #### Overview

  - What is Vitess
  - Scaling MySQL with Vitess
  - Key Concepts
- #### Getting Started

  - Run Vitess on Kubernetes
    - Custom Docker Build
  - Run Vitess Locally
- #### User Guide

  - Introduction- Client Libraries- Backing Up Data- Reparenting
        - Schema Management
        - VSchema Guide
        - Schema Swap (Tutorial)
        - Sharding
          - Horizontal Sharding (Tutorial, automated)
          - Horizontal Sharding (Tutorial, manual)
          - Sharding in Kubernetes (Tutorial, automated)
          - Sharding in Kubernetes (Tutorial, manual)
        - Vitess Sequences
        - MySQL Server Protocol
        - Vitess and Replication
        - Update Stream
        - Row Based Replication
        - Topology Service
        - Transport Security Model
        - Launching
          - Scalability Philosophy
          - Production Planning
          - Server Configuration
          - 2PC Guide
          - Troubleshooting
        - Upgrading
- #### Reference Guides

  - Vitess API- vtctl Commands
- #### Other Resources

  - Presentations- Blog- Roadmap
- #### Contributing

  - Contributing to Vitess- GitHub Workflow- Code Reviews
- GitHub

# Sharding

- #### Overview

  - What is Vitess
  - Scaling MySQL with Vitess
  - Key Concepts
- #### Getting Started

  - Run Vitess on Kubernetes
    - Custom Docker Build
  - Run Vitess Locally
- #### User Guide

  - Introduction- Client Libraries- Backing Up Data- Reparenting
        - Schema Management
        - VSchema Guide
        - Schema Swap (Tutorial)
        - Sharding
          - Horizontal Sharding (Tutorial, automated)
          - Horizontal Sharding (Tutorial, manual)
          - Sharding in Kubernetes (Tutorial, automated)
          - Sharding in Kubernetes (Tutorial, manual)
        - Vitess Sequences
        - MySQL Server Protocol
        - Vitess and Replication
        - Update Stream
        - Row Based Replication
        - Topology Service
        - Transport Security Model
        - Launching
          - Scalability Philosophy
          - Production Planning
          - Server Configuration
          - 2PC Guide
          - Troubleshooting
        - Upgrading
- #### Reference Guides

  - Vitess API- vtctl Commands
- #### Other Resources

  - Presentations- Blog- Roadmap
- #### Contributing

  - Contributing to Vitess- GitHub Workflow- Code Reviews

Sharding is a method of horizontally partitioning a database to store
data across two or more database servers. This document explains how
sharding works in Vitess and the types of sharding that Vitess supports.

## Overview

In Vitess, a shard is a partition of a keyspace. In turn, the keyspace
might be a partition of the whole database. For example, a database might
have one keyspace for product data and another for user data. The shard
contains a subset of records within its keyspace.

For example, if an application's "user" keyspace is split into two
shards, each shard contains records for approximately half of the
application's users. Similarly, each user's information is stored
in only one shard.

Note that sharding is orthogonal to (MySQL) replication.
A Vitess shard typically contains one MySQL master and many MySQL
slaves. The master handles write operations, while slaves handle
read-only traffic, batch processing operations, and other tasks.
Each MySQL instance within the shard should have the same data,
excepting some replication lag.

### Supported Operations

Vitess supports the following types of sharding operations:

- **Horizontal sharding:** Splitting or merging shards in a sharded keyspace
- **Vertical sharding:** Moving tables from an unsharded keyspace to
  a different keyspace.

With these features, you can start with a single keyspace that contains
all of your data (in multiple tables). As your database grows, you can
move tables to different keyspaces (vertical split) and shard some or
all of those keyspaces (horizontal split) without any real downtime
for your application.

## Range-based Sharding

Vitess uses range-based sharding to manage data across multiple shards.
(Vitess can also support a custom sharding scheme.)

In range-based sharding, each record in a keyspace is associated with
a sharding key that is stored with the record. The sharding key value
is also the primary key for sharded data. Records with the same sharding
key are always collocated on the same shard.

**Note:** The API uses the term "keyspace ID" to refer to the sharding key.

The full set of shards covers the range of possible sharding key values.
To guarantee a balanced use of shards, the sharding scheme should
ensure an even distribution of sharding keys across the keyspace's
shards. That distribution makes it easier to reshard the keyspace
at a later time using a more granular division of sharding keys.

Vitess calculates the sharding key or keys for each query and then
routes that query to the appropriate shards. For example, a query
that updates information about a particular user might be directed to
a single shard in the application's "user" keyspace. On the other hand,
a query that retrieves information about several products might be
directed to one or more shards in the application's "product" keyspace.

### Key Ranges and Partitions

Vitess uses key ranges to determine which shards should handle any
particular query.

- A **key range** is a series of consecutive sharding key values. It
  has starting and ending values. A key falls inside the range if
  it is equal to or greater than the start value and strictly less
  than the end value.
- A **partition** represents a set of key ranges that covers the entire
  space.

When building the serving graph for a keyspace that uses range-based
sharding, Vitess ensures that each shard is valid and that the shards
collectively constitute a full partition. In each keyspace, one shard
must have a key range with an empty start value and one shard, which
could be the same shard, must have a key range with an empty end value.

- An empty start value represents the lowest value, and all values are
  greater than it.
- An empty end value represents a value larger than the highest possible
  value, and all values are strictly lower than it.

Vitess always converts sharding keys to byte arrays before routing
queries. The value [ 0x80 ] is the middle value for sharding keys.
So, in a keyspace with two shards, sharding keys that have a byte-array
value lower than 0x80 are assigned to one shard. Keys with a byte-array
value equal to or higher than 0x80 are assigned to the other shard.

Several sample key ranges are shown below:

```
Start=[], End=[]: Full Key Range
Start=[], End=[0x80]: Lower half of the Key Range.
Start=[0x80], End=[]: Upper half of the Key Range.
Start=[0x40], End=[0x80]: Second quarter of the Key Range.
Start=[0xFF00], End=[0xFF80]: Second to last 1/512th of the Key Range.
```

Two key ranges are consecutive if the end value of one range equals the
start value of the other range.

### Shard Names in Range-Based Keyspaces

In range-based, sharded keyspaces, a shard's name identifies the start
and end of the shard's key range, printed in hexadecimal and separated
by a hyphen. For instance, if a shard's key range is the array of bytes
beginning with [ 0x80 ] and ending, noninclusively, with [ 0xc0], then
the shard's name is `80-c0`.

Using this naming convention, the following four shards would be a valid
full partition:

- -40
- 40-80
- 80-c0
- c0-

Shards do not need to handle the same size portion of the key space. For example, the following five shards would also be a valid full partition, albeit with a highly uneven distribution of keys.

- -80
- 80-c0
- c0-dc00
- dc00-dc80
- dc80-

## Resharding

In Vitess, resharding describes the process of updating the sharding
scheme for a keyspace and dynamically reorganizing data to match the
new scheme. During resharding, Vitess copies, verifies, and keeps
data up-to-date on new shards while the existing shards continue to
serve live read and write traffic. When you're ready to switch over,
the migration occurs with only a few seconds of read-only downtime.
During that time, existing data can be read, but new data cannot be
written.

The table below lists the sharding (or resharding) processes that you
would typically perform for different types of requirements:

| Requirement | Action |
| --- | --- |
| Uniformly increase read capacity | Add replicas or split shards |
| Uniformly increase write capacity | Split shards |
| Reclaim overprovisioned resources | Merge shards and/or keyspaces |
| Increase geo-diversity | Add new cells and replicas |
| Cool a hot tablet | For read access, add replicas or split shards. For write access, split shards. |

### Filtered Replication

The cornerstone of resharding is replicating the right data. Vitess
implements the following functions to support filtered replication,
the process that ensures that the correct source tablet data is
transferred to the proper destination tablets. Since MySQL does not
support any filtering, this functionality is all specific to Vitess.

1. The source tablet tags transactions with comments so that MySQL binlogs
   contain the filtering data needed during the resharding process. The
   comments describe the scope of each transaction (its keyspace ID,
   table, etc.).
2. A server process uses the comments to filter the MySQL binlogs and
   stream the correct data to the destination tablet.
3. A client process on the destination tablet applies the filtered logs,
   which are just regular SQL statements at this point.

### Additional Tools and Processes

Vitess provides the following tools to help manage range-based shards:

- The vtctl command-line tool supports
  functions for managing keyspaces, shards, tablets, and more.
- Client APIs account for sharding operations.
- The MapReduce framework
  fully utilizes key ranges to read data as quickly as possible,
  concurrently from all shards and all replicas.

## Custom Sharding

If your application already supports sharding or if you want to control
exactly which shard handles each query, Vitess can support your custom
sharding scheme. In that use case, each keyspace has a collection of
shards, and the client code always specifies the shard to which it is
directing a query.

One example of a custom sharding scheme is lookup-based sharding. In
lookup-based sharding, one keyspace is used as a lookup keyspace, and
it contains the mapping between a record's identifying key and the name
of the record's shard. To execute a query, the client first checks the
lookup table to locate the correct shard name and then routes the query
to that shard.

In a custom sharding scheme, shards can use any name you choose, and
they are always addressed by name. The vtgate API calls to use are
`ExecuteShard`, `ExecuteBatchShard`, and
`StreamExecuteShard`. None of the API calls for
**KeyspaceIds**, **KeyRanges**, or **EntityIds** are compatible with
a custom sharding scheme. Vitess' tools and processes for automated
resharding also do not support custom sharding schemes.

If you use a custom sharding scheme, you can still use the
MapReduce framework
to iterate over the data on multiple shards.

Contact: vitess@googlegroups.com  **·**  
Announcements  **·**  
© 2017 Vitess powered by Google Inc
